# Novel Mouse Model of Murine Cytomegalovirus–Induced Adaptive NK Cells

**DOI:** 10.4049/immunohorizons.2100113

**Published:** 2022-01-14

**Authors:** Isaac J. Jensen, Matthew D. Martin, Sandeep K. Tripathy, Vladimir P. Badovinac

**Affiliations:** * Department of Microbiology and Immunology, Columbia University Irving Medical Center, New York, NY;; † Department of Urology, University of Minnesota, Minneapolis, MN;; ‡ Department of Medicine, Gastroenterology Division, Washington University School of Medicine, St. Louis, MO;; § Department of Pathology, University of Iowa, Iowa City, IA;; ¶ Interdisciplinary Graduate Program in Immunology, University of Iowa, Iowa City, IA

## Abstract

NK cells are important mediators of viral control with the capacity to form adaptive immune features following infection. However, studies of infection-induced adaptive NK cells require adoptive cell transfer to lower the precursor frequency of “Ag-specific” NK cells, potentially limiting the diversity of the NK cell response. In seeking an unmanipulated model to probe the adaptive NK cells, we interrogated a wide range of Collaborative Cross (CC) inbred mice, inbred mouse strains that exhibit broad genetic diversity across strains. Our assessment identified and validated a putative “ideal” CC strain, CC006, which does not require manipulation to generate and maintain adaptive NK cells. Critically, CC006 mice, in contrast to C57BL/6 mice, are capable of developing enhanced NK cell–mediated protective responses to murine CMV infection following m157-mediated vaccination. This work both furthers our understanding of adaptive NK cells and demonstrates the utility of CC mice in the development and interrogation of immunologic models.

## INTRODUCTION

NK cells are critical early mediators of viral control through cytokine production and cytolytic activity. In lieu of rearranged Ag-specific receptors, NK cells recognize infected, damaged, and cancerous cells through a complex array of activating and inhibitory receptors. These receptors recognize a variety of ligands, including those that are host derived [e.g., MHC (1)] as well as some that are pathogen derived [e.g., m157 expressed by murine CMV (MCMV) (2, 3)]. These receptor–ligand interactions tune NK cell activity through licensing under homeostatic conditions (4) and the acquisition of enhanced or adaptive qualities following pathogen encounter/vaccination (5, 6).

With respect to the acquisition of adaptive immune features following infection, the interaction between Ly49H^+^ NK cells from C57BL/6 (B6) mice with the m157 viral immunoevasin expressed by MCMV is among the best characterized (7), and this system is broadly analogous to the interaction between NKG2C^+^ NK cells with human CMV (8). These adaptive NK cells undergo extensive numeric expansion with differentiation to become predominantly CD11b^+^CD27^−^KLRG1^+^ terminally differentiated cells with increased Ly49H expression, cytokine production, cytolytic activity, and capacity to provide host protection (6). However, this system relies on the transfer of a few Ly49H-expressing NK cells into hosts that lack the receptor, thereby lowering the precursor frequency of “Ag-specific” cells for MCMV (6). Given the wide variety of receptors that contribute to NK cell interactions with target cells and the influence of varied expression by these numerous receptors on NK cell–target interactions, the transfer of a small number of NK cells selects against this natural variation enriching the most avid responders (9). The influence of this variation is observed in wild-type B6 mice wherein NK cells do respond to MCMV infection and undergo numeric expansion with maturation, although they rapidly wain such that adaptive NK cells are no longer distinguishable less than a month postinfection, relative to non-infected hosts (10). Confounding this issue further is the lack of expression of Ly49H in most other common inbred mouse strains such that suitable alternatives are not readily apparent (11).

Notably, the Collaborative Cross (CC) model derives multitudes of inbred mouse strains through a funnel breeding strategy, utilizing five classical inbred strains (A/J, B6, 129S1/SvImJ, NOD/ShiLtJ, and NZO/HILtJ) and three wild-derived strains (CAST/EiJ, PWK/PhJ, and WSB/EiJ) (12). This resource generates a wide array of genetic diversity across the mouse strains and enables the identification/interrogation of genetic factors that underlie complex phenotypic traits as well as the potential for the identification of novel model strains for experimental interrogation. In this study we demonstrate the latter through the identification of a CC mouse strain [CC006/TauUncJ (CC006)] as an ideal model for the study of unmanipulated adaptive NK cells generated following interaction of Ly49H with m157. Specifically, we demonstrate the range of Ly49H expression across multiple CC strains and the rationale in choosing CC006 as a candidate for in-depth analysis. Following MCMV infection, Ly49H NK cells from CC006 mice exhibit enhanced expansion relative to B6 mice and maintain a discernable adaptive phenotype. Importantly, vaccination with m157-expressing target cells promotes the formation of adaptive NK cells and enhances MCMV control in CC006 mice but not in B6 mice. These results both enhance our understanding of adaptive NK cells and accentuate the utility of CC mice in probing immunologic questions in the future.

## MATERIALS AND METHODS

### Ethics statement

Experimental procedures using mice were approved by the University of Iowa Animal Care and Use Committee under Animal Care and Use Review Form protocol numbers 6121915 and 9101915. The experiments performed followed Office of Laboratory Animal Welfare guidelines and the Public Health Service Policy on Humane Care and Use of Laboratory Animals. Cervical dislocation was used as the euthanasia method for all experimental mice.

### Mice

Inbred B6 and BABL/c mice were purchased from the National Cancer Institute (Frederick, MD) and maintained in the animal facilities at the University of Iowa at the appropriate biosafety level. B6 mice with transgenic expression of m157 (B6-m157) were described previously (13, 14). Female CC mice were obtained from the Systems Genetics Core Facility at the University of North Carolina, Chapel Hill (15) as previously described (16). CC006 mice were subsequently bred and maintained at the University of Iowa under specific pathogen-free conditions at the appropriate biosafety level and used at 6–20 wk of age. All animal experiments were approved by the Institutional Animal Care and Use Committee of the University of Iowa. Ab-mediated cell depletion was performed as previously described (17).

### CC chromosomal inheritance and breeder information

Information for CC strains is available at UNC Systems Genetics (http://csbio.unc.edu/CCstatus/index.py). Inheritance of *Klra8*, *H2-D1*, *H2-K1*, *H2-M1*, *H2-Ab1*, *H2-Eb1*, *H2-Q6*, and *H2-T23* was determined by comparing the chromosomal regions for the indicated genes with the available genetic information for the respective CC strains.

### Infections

Mice were infected i.p. with 10^4^ PFU of MCMV-Smith. Challenge studies used an infectious dose of 10^5^ PFU. MCMV viral titers were quantified using a plaque assay on M2–10B4 cells, as previously described (18).

### Splenocyte transfer

Splenocytes from either B6-m157 or littermate control B6 mice were isolated, and 10^6^ cells were injected by i.v. tail vein injection into the respective immunization groups for CC006 and B6 recipients.

### Tissue harvest

Tissue collection was performed as previously described (13).

### Flow cytometry

Flow cytometry was performed as previously described (17). The following mAb clones were used: eBioscience [NK1.1 (PK136), CD3 (17A2), NKp46 (29A1.4), CD122 (TM-b1), IFN-γ (XMG1.2)] and BioLegend [Ly49H (3D10), Ly49D (4E5), KLRG1 (2F1/KLRG1), and CD62L (MEL-14)].

### In vitro stimulation

Plate-bound Ab stimulation was performed as described (13). Alternately, splenocytes were incubated at 37°C with 20 ng/ml each of rIL-12 and rIL-18 (R&D Systems) for 8 h with brefeldin A added during the last 4 h of stimulation.

### Statistical analysis

Data were analyzed using Prism 8 software (GraphPad Software) using a two-tailed Student *t* test (for two individual groups; when variance was unequal, then a Mann–Whitney *U* test was used), one-way ANOVA with a Bonferroni post hoc test (for more than two individual groups; when variance was unequal, then a Kruskal–Wallis test with a Dunn’s post hoc test was used), or two-way ANOVA (for multiparametric analysis of two or more individual groups; pairing was used for samples that came from the same animal) with a confidence interval of >95% to determine significance (*p* < 0.05). Data are presented as SEM.

## RESULTS

**FIGURE 1.**
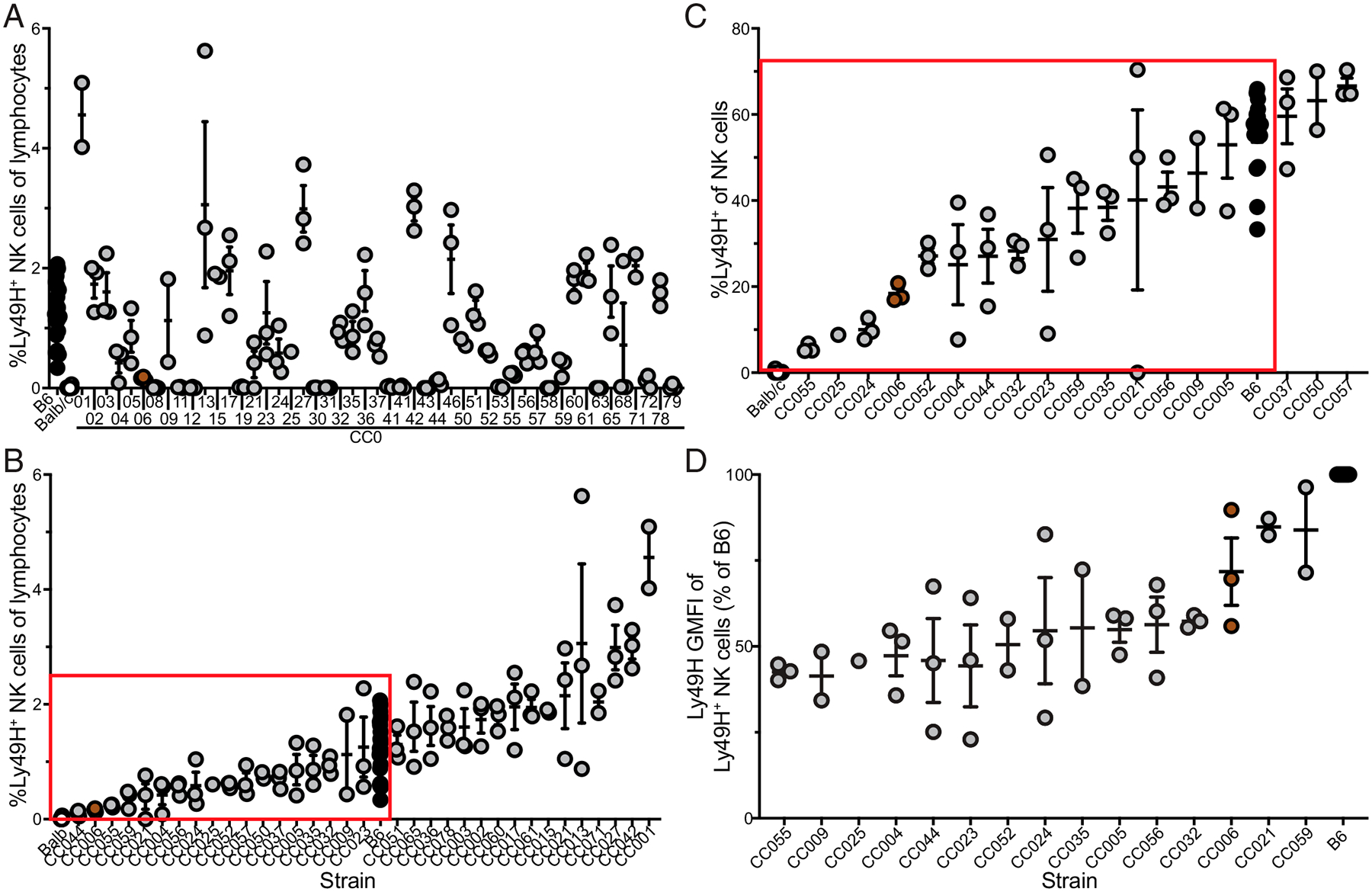
Diversity in Ly49H expression among CC006 mice. (**A**–**C**) The frequency of (A) Ly49H NK cells among lymphocytes, (B) Ly49H NK cells among lymphocytes (excluding CC mice lacking Ly49H, ordered from least to greatest, and (C) Ly49H^+^ among NK cells (among those boxed in B, ordered from least to greatest) in the PBLs of B6, BALB/c, and CC mice. (**D**) Ly49H geometric mean fluorescence intensity (GMFI) by Ly49H^+^ NK cells (among those boxed in C, ordered from least to greatest). Data are from one to three individual experiments with 1–20 mice per group. Error bars represent SEM.

**FIGURE 2.**
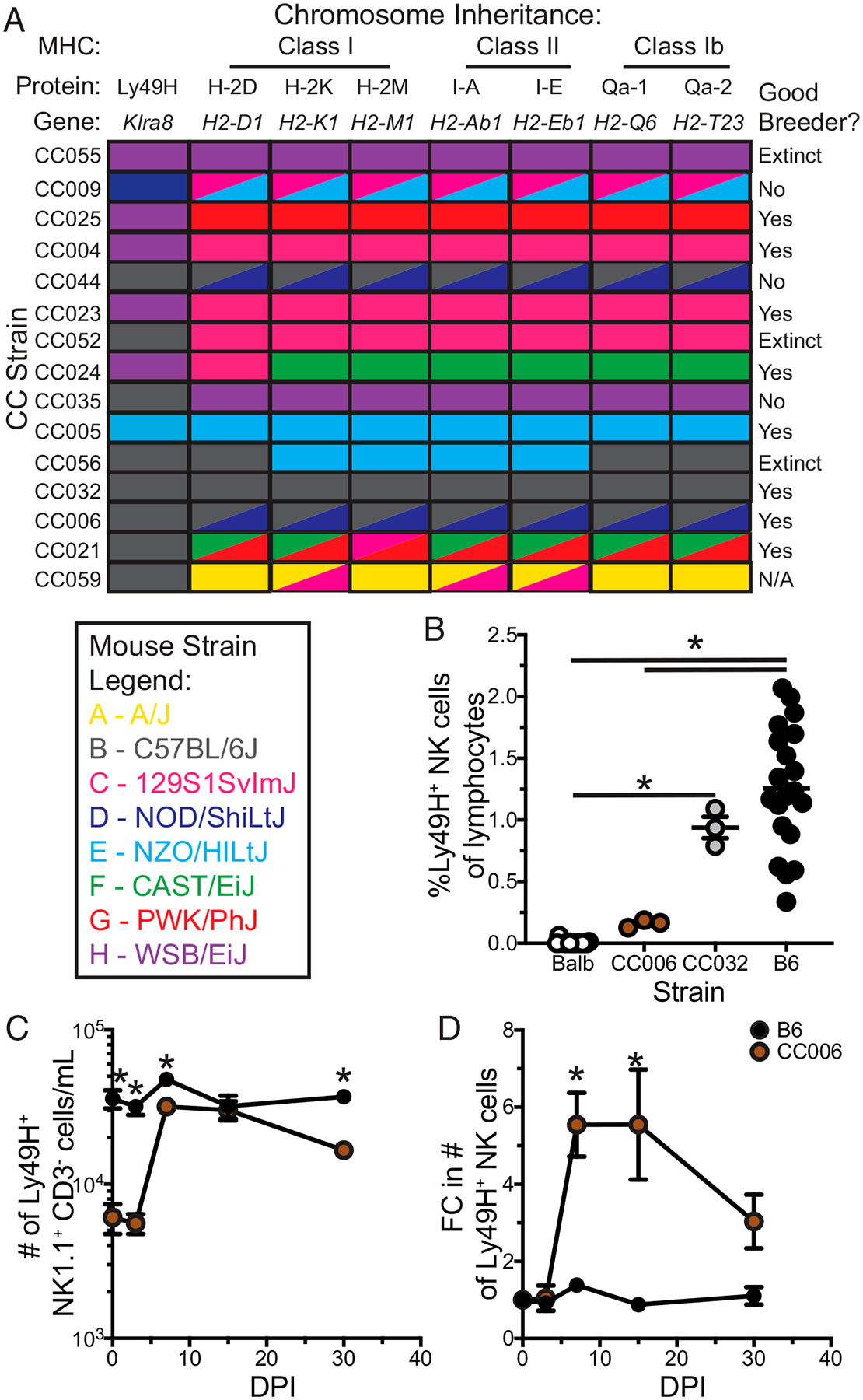
Selection of CC006 model for MCMV infection. (**A**) Inheritance of Ly49H, MHC class I, MHC class II, MHC class Ib, and breeder information for CC strains identified in Fig. 1D. (**B**) Frequency of Ly49H NK cells among lymphocytes for B6, BALB/c, CC006, and CC032 mice. (**C** and **D**) The number (C) and fold change (D) in the number (relative to prior to infection) of Ly49H NK cells per milliliter of blood at days 0, 3, 7, 15, and 30 after MCMV infection in B6 and CC006 mice. Data are representative of three independent experiments with 3–20 mice per group. Error bars represent SEM. *p < 0.05.

To identify an unmanipulated mouse strain for probing adaptive NK cells, we began by operating under the assumption that the reason detection of adaptive NK cells is blunted in B6 mice is due to the high starting number of Ly49H^+^ NK cells (“overcrowding”) and high initial expression of Ly49H on NK cells in B6 mice. We therefore established a set of optimal criteria for a hypothetic ideal mouse strain relative to a B6 mouse: reduced frequency (but still some) Ly49H NK cells among lymphocytes (Fig. 1A, 1B), reduced frequency of Ly49H^+^ cells among NK cells (Fig. 1C), and reduced Ly49H expression per cell (Fig. 1D). This yielded a preliminary list of 15 strains. To ensure that our observations would not be hindered by variations in the origin of the Ly49H receptor or the licensing of the NK cells, mice were further selected for those strains that derived their Ly49H, H-2D/K/M, I-A/E, and Qa-1/2 loci from B6 mice (Fig. 2A). Notably, future benefit can be garnered from this MHC selection with regard to the study of T cells, as many MHC-based immunologic tools have been built around the use of B6 mice (16). Of the resultant three strains only CC032 and CC006 readily bred (Fig. 2A). However, in comparing CC006 to CC032 it was apparent that CC032 did not have a significant reduction in Ly49H^+^ NK cells relative to B6 mice whereas CC006 did (Fig. 2B). Therefore, CC006 was chosen as a putative strain for probing adaptive features of NK cells. Although they share some critical loci with B6 mice, CC006 mice are distinct with a different coat color and genetics derived from all eight founder strains (Supplemental Fig. 1A, 1B). However, as mentioned, *Klra8* (the gene that encodes Ly49H) is derived from B6 mice, as are several other NK cell receptor genes [e.g., *Klrb1* (NK1.1) and *Klra9* (Ly49D)] (Supplemental Fig. 1C). This demonstrates how even a large number a priori decisions can still be met through the genetic variability afforded by CC mouse strains.

When the expansion of Ly49H NK cells in B6 mice with CC006 mice was compared, the utility of these selection criteria became apparent. The number of Ly49H NK cells in the PBLs was significantly lower in CC006 mice prior to infection. However, following MCMV infection the number of Ly49H NK cells expanded to reach an equivalent number of Ly49H NK cells in CC006 and B6 mice (Fig. 2C). As a consequence, Ly49H NK cells underwent a greater fold expansion in CC006 mice relative to B6 mice, and this increased expansion enabled detection of an expanded population of Ly49H NK cells 30 d postinfection (an early memory time point) (Fig 2D). Thus, Ly49H NK cells in CC006 mice have greater expansion than do those in B6 mice, likely due to overcrowding, and this provides an unmanipulated mouse strain for interrogating adaptive NK cells.

**FIGURE 3.**
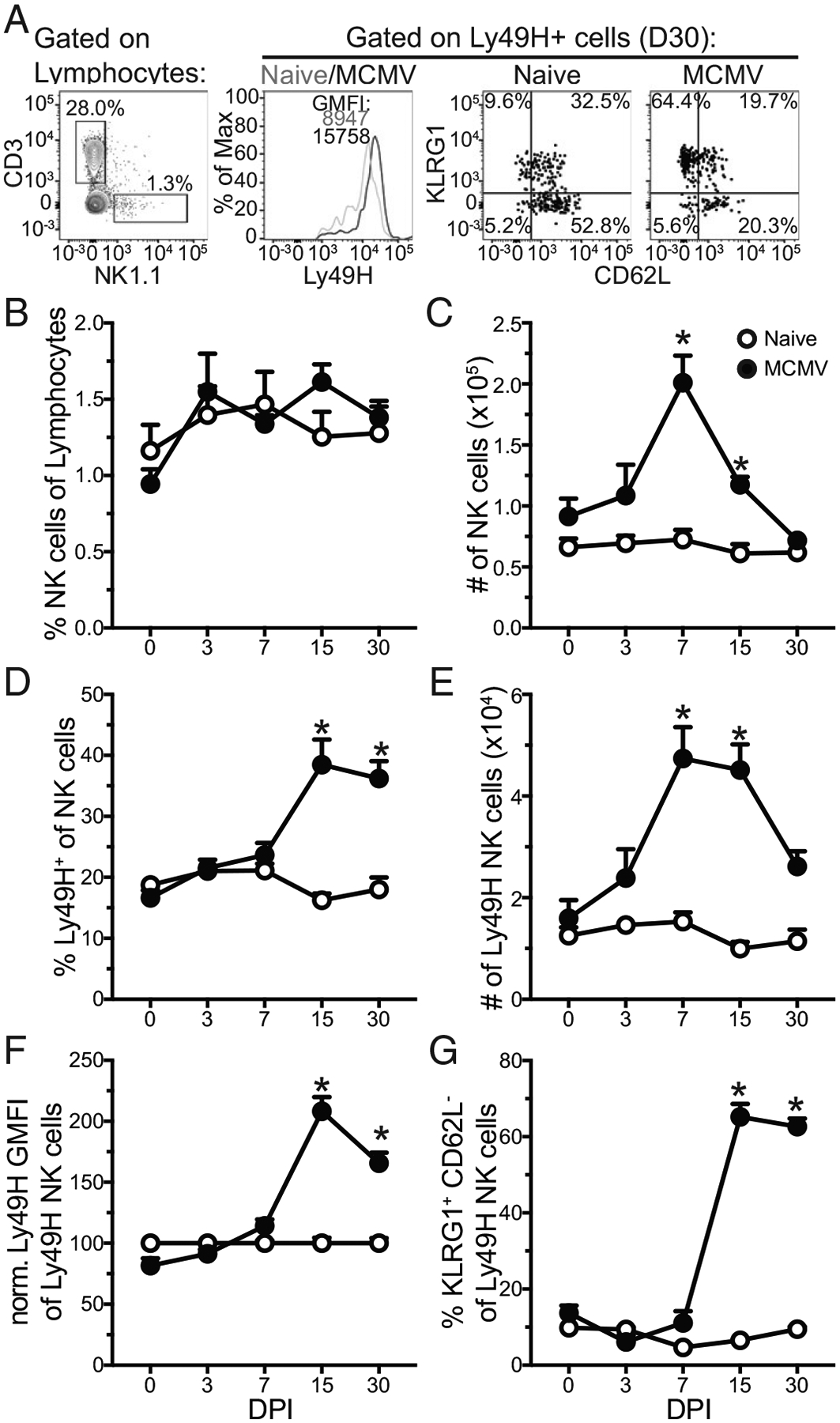
Generation and maintenance of adaptive NK cells in CC006 mice. (**A**) Representative gating for NK cells, Ly49H staining among Ly49H NK cells, and gating of KLRG1 and CD62L subsets among Ly49H NK cells. (**B**–**E**) Frequency (B and D) and number per milliliter (C and E) of NK cells (B and C) and Ly49H NK cells (D and E) in naive and MCMV infected CC006 mice at days 0, 3, 7, 15, and 30 postinfection. (**F** and **G**) Ly49H GMFI (F) and frequency (G) of KLRG1^+^CD62L^−^cells among Ly49H NK cells in naive and MCMV infected CC006 mice at days 0, 3, 7, 15, and 30 postinfection. Data are representative of three independent experiments with five mice per group. Error bars represent SEM. *p < 0.05.

To delve into whether expansion coincided with adaptive qualities, Ly49H NK cell expansion kinetics, acquisition of an adaptive phenotype, and maintenance to an early memory time point (day 30 postinfection) was interrogated in the PBLs of MCMV-infected and non-infected control (naive) CC006 mice over the course of 30 d (Fig. 3A). The representation of NK cells in the PBLs was consistent across all time points, although numeric expansion was observed at days 7 and 15 postinfection (Fig. 3B, 3C). Interestingly, there was a significant increase in the representation of Ly49H NK cells beginning 15 d postinfection (Fig. 3D), coinciding with the contraction of the NK cell response to infection (Fig. 3C). As such, the number of Ly49H NK cells remained elevated, in contrast to total NK cells, suggesting the maintenance of an adaptive NK cell population (Fig. 3E). Supporting this notion was the observation that Ly49H remained upregulated, a critical marker of adaptive NK cells, reflecting the avidity maturation of NK cells expressing the Ly49H receptor, out to 30 d postinfection (Fig. 3F) (19). To further clarify this putative adaptive NK cell population, the expression levels of KLRG1, which is upregulated on adaptive NK cells (6), and CD62L, which is downregulated on adaptive NK cells (20) and whose expression was recently shown to delineate two populations of adaptive NK cells (21), were assessed. Interestingly, we observed that the frequency of CD62L^−^KLRG1^+^ cells remained substantially increased among Ly49H NK cells even at 30 d postinfection, demonstrating the adoption and maintenance of a discrete phenotype by adaptive NK cells in CC006 mice (Fig. 3G).

To extend the notion of maintenance of an adaptive NK cell population, the representation of KLRG1^+^CD62L^−^cells among Ly49H NK cells in the PBLs, spleen, liver, inguinal lymph node, and cervical lymph node was investigated 200 d after MCMV infection. Interestingly, this putative adaptive population remained elevated in the PBLs and spleen even at this late timepoint (Supplemental Fig. 2A), further reinforcing the notion of a lasting memory response. Although a phenotypic difference can delineate populations of interest, an important characteristic of adaptive immunity is enhanced function during a recall response (6, 20). Thus, the capacity of splenic KLRG1^+^CD62L^−^Ly49H NK cells to produce IFN-γ following stimulation at 200 d post–MCMV infection was investigated. Critically, these adaptive NK cells exhibited enhanced cytokine production following stimulation of either Ly49H or Ly49D, relative to naive hosts, demonstrating enhanced functional capability (Supplemental Fig. 2B).

**FIGURE 4.**
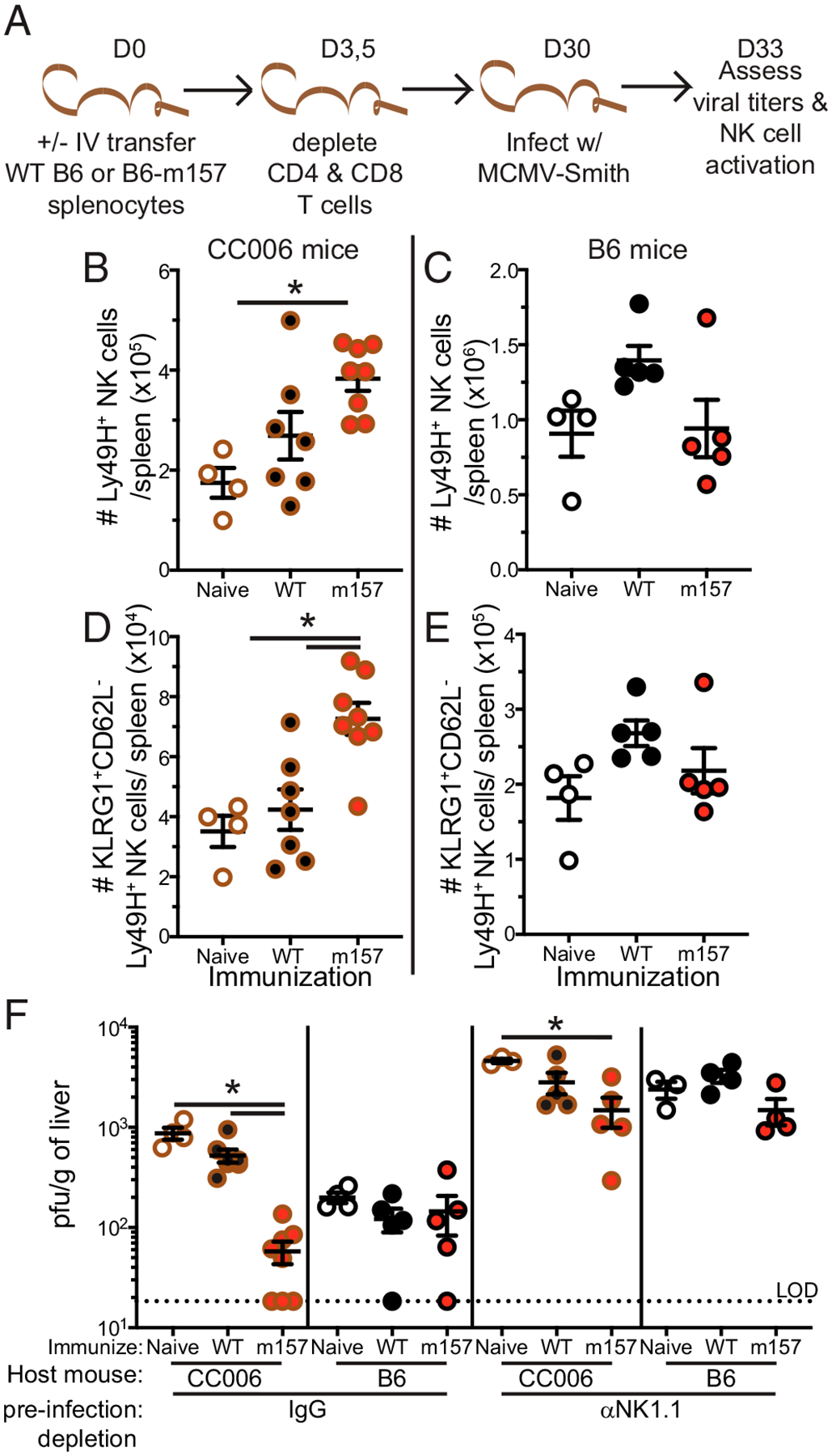
Generation of protective NK cell memory responses by immunization in CC006 mice. (**A**) Experimental design. B6 and CC006 mice received i.v. transfer of either B6 splenocytes or B6-m157–expressing splenocytes, and no transfer mice served as an additional control. At day (D)3 and D5 posttransfer, mice that received splenocyte transfer were depleted of CD4 and CD8 T cells. Mice were bled at D7 to assess the impact of immunization on Ly49H NK cells. At D30 posttransfer, mice were infected with MCMV-Smith; a set of mice from each group were depleted of NK cells both 2 d prior to and on the day of infection. NK cell activation and viral titers were assessed at D33 posttransfer (D3 MCMV postinfection). (**B**–**E**) The number of Ly49H NK cells (B and C) and KLRG1^+^CD62L^−^Ly49H NK cells (D and E) in the spleen of CC006 (B and D) and B6 (C and E) mice 3 d after MCMV infection that were either naive, control immunized, or m157 immunized. (**F**) D3 liver MCMV titers in CC006 and B6 mice that were either naive, control immunized, or m157 immunized; infectious groups also included NK-depleted controls. Samples are representative of two independent experiments with four to eight mice per group. Error bars represent SEM. *p < 0.05.

To determine whether this putative adaptive NK cell population has the capacity to provide enhanced protection during infection the following immunization strategy was used. CC006 and B6 mice received i.v. transfer of either splenocytes from B6-m157 or littermate control splenocytes (Fig. 4A). This should elicit rejection of both splenocyte populations in the CC006 mice but elicit a Ly49H-mediated adaptive response only in those that receive B6-m157 splenocytes. Conversely, only the B6-m157 splenocytes should be rejected in the B6 mice (22). Immediately following splenocyte immunization, mice were depleted of CD4 and CD8 T cells with depleting Abs to quash any adaptive immune response by non-NK cells (Fig. 4A). Because Ab-mediated depletion elicits Ab-dependent cellular cytotoxicity by NK cells, this may have the additional benefit of further priming the NK cells (23, 24), although this requires further interrogation. To ensure that this immunization protocol elicited an adaptive NK cell response, the frequency of KLRG1^+^CD62L^−^Ly49H^+^ among NK cells was evaluated in the PBLs at day 7 after splenocyte transfer. Mice receiving neither splenocyte transfer nor T cell depletion served as controls. Notably, both transfer groups elicited a response in the CC006 mice (Supplemental Fig. 3A) whereas neither transfer group responded in the B6 mice (Supplemental Fig. 3B). This likely reflects the much higher number of Ly49H NK cells in the B6 mice “washing out” the influence of the immunization and suggests that B6 mice may not benefit from this immunization. Mice were then allowed to develop memory up to day 30 postimmunization, at which point they were challenged with MCMV infection (Fig. 4A). Mice in each immunization group were also separated into two groups that received either control IgG or anti-NK1.1 depleting Ab 2 d prior to and on the day of infection. This enables the interrogation of the NK cell contribution to MCMV control (22). At day 3 postinfection (day 33 postimmunization) splenic NK cell activation and liver MCMV viral titers were assessed (Fig. 4A). NK cell depletion was confirmed in splenic NK cells (Supplemental Fig. 3C, 3D).

Notably, CC006 mice that received B6-m157 immunization had more total Ly49H NK cells and KLRG1^+^CD62L^−^Ly49H NK cells compared with both naive and B6 immunized hosts (Fig. 4B, 4D), indicating an enhanced recall response following infection. However, no enhanced recall response was observed in B6 mice (Fig. 4C, 4E), consistent with the lack of response to immunization (Supplemental Fig. 3B). Critically, CC006 mice with B6-m157 immunization exhibited enhanced control of MCMV infection relative to naive hosts and B6 immunized hosts (Fig. 4F). Furthermore, B6 mice exhibited no difference in MCMV control regardless of immunization status, further consistent with the lack of response to immunization. Importantly, mice depleted of NK cells did not robustly control MCMV infection, although it is notable that m157 immunized CC006 mice that were depleted of NK cells still exhibited some degree of enhanced protection. A potential explanation for this discrepancy is the presence of a small, but still detectable, number of NK cells in the NK-depleted hosts (Supplemental Fig. 3C), and thus these remaining adaptive NK cells may still be able to exert some degree of improved viral control. Cumulatively, this demonstrates that B6-m157 immunization in CC006 mice provides NK cell–mediated control of MCMV infection and elicits the formation of adaptive NK cells.

## DISCUSSION

This work presents a novel unmanipulated mouse model in which to study adaptive NK cell responses. Additionally, it builds on the identification of adaptive NK cells for their potential delineation in other model systems. Future investigations should be performed to delve deeper into the distribution of these adaptive NK cells throughout the host to delineate whether the population interrogated in this study are also localized within tissues or whether other adaptive NK cell populations exist. Further demonstration of changes in morbidity and mortality as a consequence of NK cell memory should be investigated in this model system. Related to the former two points of interest is the possibility that the KLRG1^+^CD62L^−^population, which was emphasized in our study, may represent only one potential adaptive NK cell population (possibly analogous to effector memory T cells with which they share a phenotype). Thus, multiple adaptive NK cell populations may exist (e.g., a central memory-like population that expresses CD62L) or the population of KLRG1^+^CD62L^−^NK cells encompasses several discrete adaptive NK cell populations. Identification of putative memory NK cell populations should also be interrogated in a diverse array of mouse strains including both conventional inbred strains (such as B6 mice) and other CC strains. These analyses can be paired with the development of T cell and B cell memory to develop a comprehensive understanding of coordination of adaptive lymphocytes.

However, a diverse assortment of CC mice exists with a large variation in NK cell Ly49H populations and unique MHC signatures from the different founders, and these are likely to fuel their own interesting interrogations. Such interrogation may provide insights into the development of adaptive NK cells in humans with a much broader range of genetic variability, relative to inbred mice. These investigations should incorporate numeric assessment of NK cells across tissues and may be further extended through the breeding of F_1_ mice with either strains of interest or some of the classical inbred strains, as has been previously investigated (25). These analyses may elucidate the molecular underpinnings for variation in the baseline differences in NK cell number and expression of NK cell receptors such as Ly49H. Our work therefore stands as a testament of the possibilities open to interrogation by highlighting the utility of CC mice in the formulation and exploration of immunologic models. This further extends beyond NK cells to any other immunologic questions of interest posed, as this resource continues to extend.

## Supplementary Material

1

## Abbreviations used in this article:

B6: C57BL/6
B6-m157: B6 mice with transgenic expression of m157
CC: Collaborative Cross
CC006: CC006/TauUncJ
MCMV: murine CMV
