# Supplementary material for: Novel Mouse Model of Murine Cytomegalovirus–Induced Adaptive NK Cells
Source: Immunohorizons. Author manuscript; Available in PMC 2022 Nov 5. (PMC9636593; doi:10.4049/immunohorizons.2100113)
Supplement: 1 [file NIHMS1846483-supplement-1.pdf]

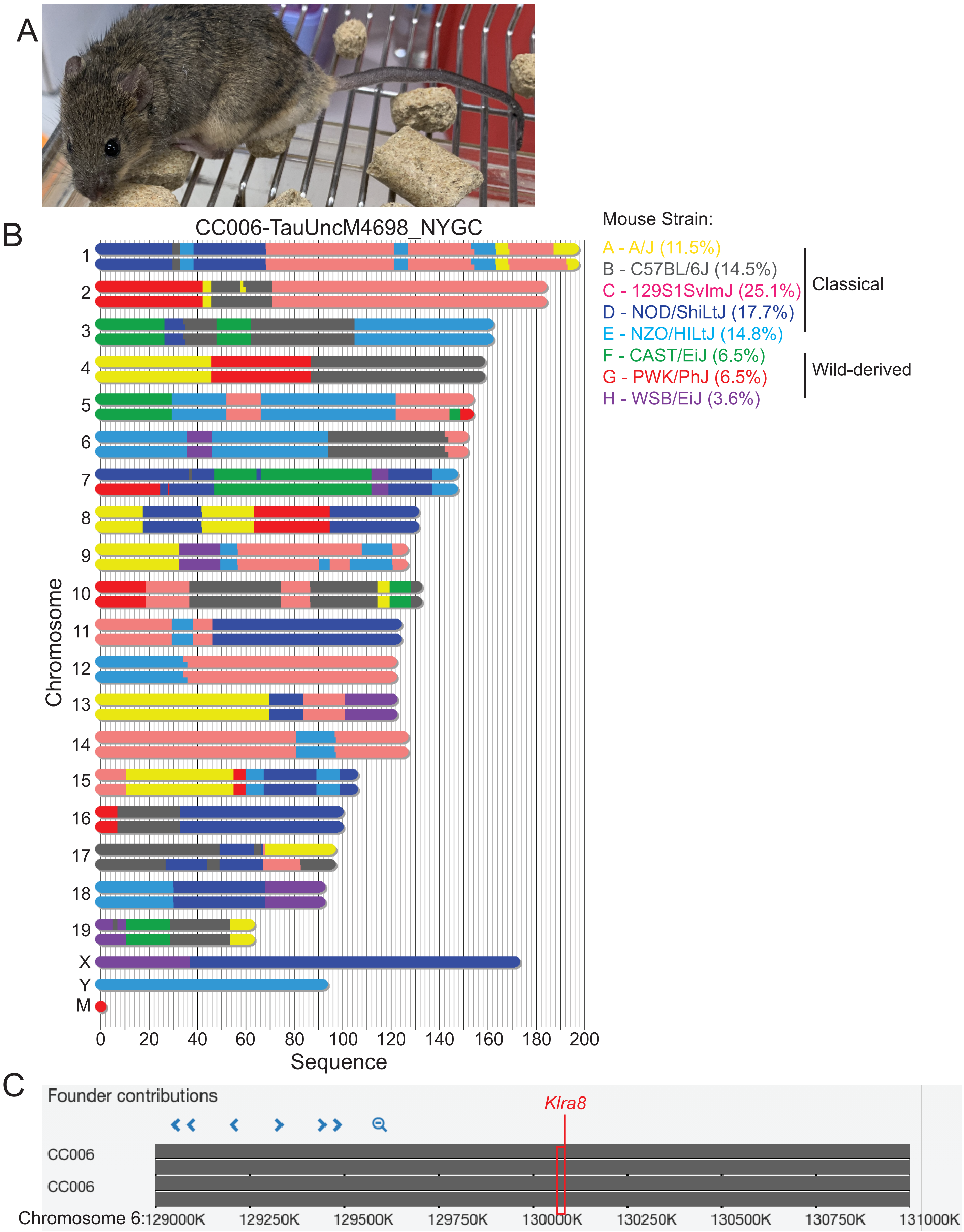

Supplemental Figure 1: Phenotype and genotype of CC006 mice. (**A**) Image of CC006 mouse. (**B**) Genetic founder composition of CC006 mice. (**C**) Genetic founder composition of Ly49H locus in CC006 mice.

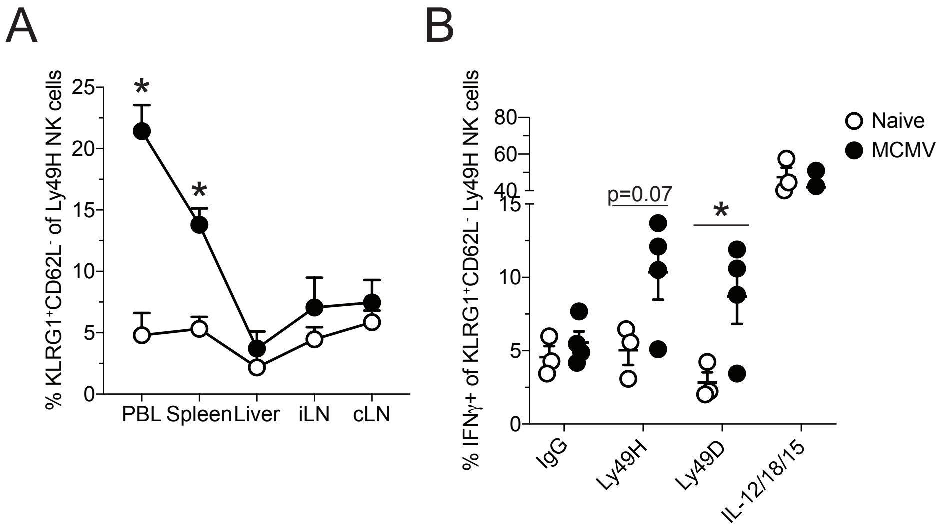

Supplemental Figure 2: Long-term maintenance and function of adaptive NK cells in CC006 mice. The frequency of (**A**) KLRG1^{+} CD62L^{-} cells among Ly49H NK cells [in the PBL, spleen, liver, iLN, and cLN] and (**B**) IFNg^{+} of splenic KLRG1^{+} CD62L^{-} Ly49H NK cells [following stimulation with control IgG, aLy49H, aLy49D, and IL-12+IL-18+IL-15] at D200 post-infection MCMV infected and age-matched naïve mice. Data are from a single experiment with 3-4 mice per group. Error bars represent standard error of the mean. *=p-value<0.05.

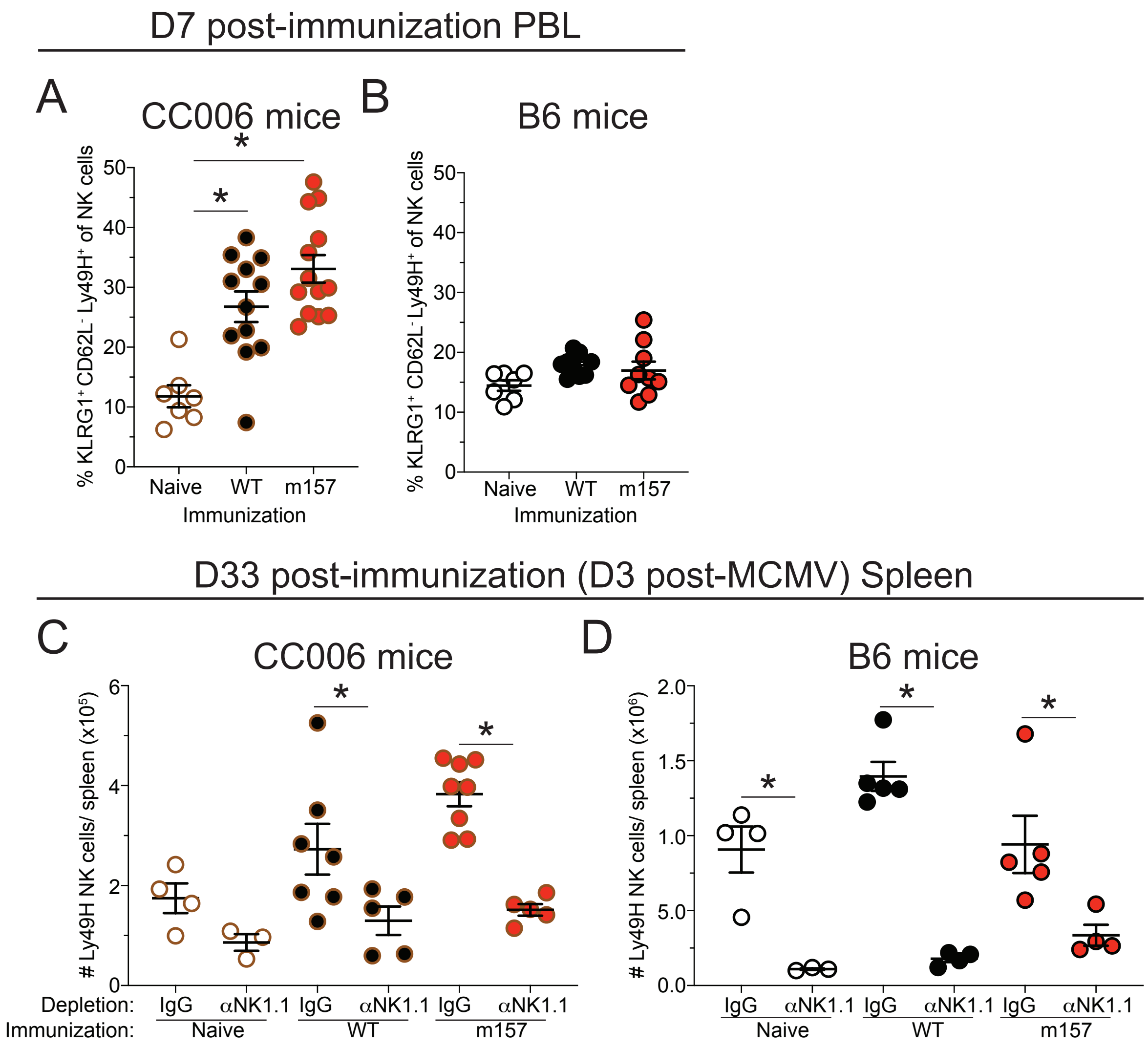

Supplemental Figure 3: Assessment of NK cell activation following immunization and determination of depletion during MCMV challenge. The frequency of $KLRG1^{+}CD62L^{-}$ $Ly49H^{+}$ cells among NK cells at D7 post-immunization in naïve, B6 immunized, and B6-m157 immunized (**A**) CC006 and (**B**) B6 mice. The number of Ly49H NK cells per spleen of NK depleted and non-depleted naïve, B6 immunized, and B6-m157 immunized (**A**) CC006 and (**D**) B6 mice. Data are from a 1-2 experiments with 3-13 mice per group. Error bars represent standard error of the mean. *=p-value<0.05.
